# Fine-Scale Distribution and Spatial Variability of Benthic Invertebrate Larvae in an Open Coastal Embayment in Nova Scotia, Canada

**DOI:** 10.1371/journal.pone.0106178

**Published:** 2014-08-25

**Authors:** Rémi M. Daigle, Anna Metaxas, Brad deYoung

**Affiliations:** 1 Department of Oceanography, Dalhousie University, Halifax, Nova Scotia, Canada; 2 Department of Physics and Physical Oceanography, Memorial University, St. John’s, Newfoundland, Canada; Simon Fraser University, Canada

## Abstract

This study quantified the fine- scale (0.5 km) of variability in the horizontal distributions of benthic invertebrate larvae and related this variability to that in physical and biological variables, such as density, temperature, salinity, fluorescence and current velocity. Larvae were sampled in contiguous 500-m transects along two perpendicular 10-km transects with a 200-µm plankton ring net (0.75-m diameter) in St. George’s Bay, Nova Scotia, Canada, in Aug 2009. Temperature, conductivity, pressure and fluorescence were measured with a CTD cast at each station, and currents were measured with an Acoustic Doppler Current Profiler moored at the intersection of the 2 transects. Gastropod, bivalve and, to a lesser extent, bryozoan larvae had very similar spatial distributions, but the distribution of decapod larvae had a different pattern. These findings suggest that taxonomic groups with functionally similar larvae have similar dispersive properties such as distribution and spatial variability, while the opposite is true for groups with functionally dissimilar larvae. The spatial variability in larval distributions was anisotropic and matched the temporal/spatial variability in the current velocity. We postulate that in a system with no strong oceanographic features, the scale of spatially coherent physical forcing (*e.g.* tidal periodicity) can regulate the formation or maintenance of larval patches; however, swimming ability may modulate it.

## Introduction

The dynamics and persistence of populations of marine benthic invertebrates are affected by connectivity, which is in turn regulated by dispersal during the planktonic larval phase [1], [2]. For example, spatial and temporal variability in larval supply and settlement influences the patterns of recruitment of new individuals to adult populations [3], [4]. The scale of temporal variability in larval abundance can range from decadal climatic variations [5] to diel or tidal vertical migrations [6]. Spatially, variability in larval abundance can result from the variation in water properties at regional scales [7] to small scale features of the water column that may aggregate and transport invertebrate larvae, such as up- or downwelling [8], [9], internal tidal bores [10], or frontal systems and Langmuir cells [11]. Many different physical processes can influence the spatial or temporal pattern of larval dispersal and connectivity. At large spatial or temporal scales (km or y), the variability in larval abundance leads to spatially dependant variability in recruitment, and consequently, spatially dependant species assemblages. At smaller scales (m or h), physical processes aggregate larvae and alter larval transport, ultimately affecting larval dispersal. Additionally, patchiness or spatial heterogeneity generated by variability in larval abundance, affects the perceived patterns in the distribution of planktonic larvae, and may contribute to the detection of erroneous patterns through spatial aliasing or masking of the true pattern by high sampling variability [11].

Quantifying larval dispersal and connectivity is difficult since larvae, while numerous, are distributed patchily and are generally difficult to sample accurately [1], [2]. Traditional net sampling provides estimates of abundance at one point in time and space, but does not provide information on areas of larval release, or potential settlement. Geochemical tracers and genetic markers have been used to quantify realized dispersal, but bio-physical modelling remains the only method currently used to predict trajectories of larval dispersal for all species. There are examples where the dispersal of relatively large, short-lived tunicate larvae has been visually tracked [12], but this technique is not practical for very small or long lived larvae. Most biophysical models are based on either numerical circulation models or advection-diffusion models used to quantify the effects of the physical properties of the ocean on larval dispersal [13]. The validity of the bio-physical model is limited by the model’s ability to resolve relevant physical features of the water column with which larvae could interact behaviourally, such as up- or downwelling, as well as by the incorporation of this behaviour into the “bio” component of the bio-physical model. Studying patterns of larval distribution and their mechanisms of formation at different scales [14], [15] enable development of valuable parameterization of the biological components of these models.

Biological components typically incorporated in models, such as longer pelagic larval duration (PLD), mortality, and vertical migration, can have a significant impact on larval dispersal [6], [16]–[19]. Larvae with longer PLDs may be advected further, and be associated with longer dispersal distances. However, observations of the spread of invasive species, genetic studies of population structure, direct observations, and experimental estimates of dispersal distances show that this is not always true [19], [20]. Advection does depend on larval duration, but is also influenced by larval behaviour, larval characteristics (*e.g.* swimming ability) and the local ocean current regime. Species with short PLDs can be associated with a surprisingly homogeneous population structure and species with longer PLDs can be retained locally [19], [20]. Some of this variability may be explained by the biases related to the quantification methods; however, there is certainly variability in the relationship between PLD and dispersal distance that can be attributed to larval behaviour and interactions with oceanographic features. An example of such a behavioural interaction is selective tidal stream transport [6], [21], [22], where larvae of certain species migrate vertically with the same periodicity as the tidal cycle, towards the surface at flood tide and towards the sea floor at ebb tide, taking advantage of the differential transport to reach the head of the estuary.

By comparing the spatial scale in larval patchiness with that of the physical variables of the water column (temperature, salinity etc.), we can determine the relative contribution of different physical features (upwelling, internal tidal bore etc.) in generating patterns in larval abundance. For example, a similarity between the scale of larval patch size and the variability in temperature, but not of salinity, implies that temperature is more important in determining the larval pattern. Both a correlation in space between larval abundance and physical variables of the water column, and a similarity in the scale of spatial variability for biological and physical variables can be used as indicators of the physical features that regulate larval abundance [23]. In this study, we determined the patch size through spatial autocorrelation in larval abundance of different taxonomic groups (bryozoans, bivalves, gastropods, decapods) and in the physical variables of the water column (temperature, salinity, fluorescence, density, depth of the fluorescence maximum and depth of the mixed layer), and identified relevant physical or biological processes that may influence larval distributions at fine spatial scales (0.5 km).

### Bay-scale larval distribution

In a concurrent study [24], we investigated mechanisms of pattern formation in the larval distributions of benthic invertebrates by relating the spatial and temporal variability in the larval distributions to that of physical and biological variables at scales of 10 s km’s. At bay-wide scales, gastropod, bivalve and, to a lesser extent, bryozoan larvae had very similar spatial distributions, but the distribution of decapod larvae followed a different pattern. These results suggested that taxonomic groups that have functionally (i.e. swimming ability) similar larvae (e.g. bivalves and gastropods) also show similar dispersion properties (distribution and spatial variability), while the opposite is true for groups with functionally dissimilar larvae (e.g. bivalves and decapods). Patterns in observed horizontal distribution at the broad scale were determined during the larval phase, and that the primary mechanism for pattern formation was larval interaction with physical oceanographic structures (e.g. stratification and tidal currents).

### Physical Characteristics of St. George’s Bay

St. George’s Bay is an open coastal embayment, ∼45 by 45 km, generally shallow, with a mean depth of about 20 m and a maximum depth of 35–40 m. There is little freshwater runoff into the Bay, and the primary forcing of the circulation is from the tides and wind, both locally and indirectly from the neighbouring Gulf of St. Lawrence. The tidal currents in the Bay are generally weak mixed diurnal to semidiurnal, with a tidal range of about 1.5 m (Canadian Hydrographic Service, water.charts.gc.ca/twl-mne/index-eng.asp). The dominant tidal constituent, the semi-diurnal M_2_ velocity has an amplitude near the mouth of ∼0.10 m s^−1^. Other tidal constituents are much smaller.

There is relatively weak horizontal spatial structure in the density field, and essentially no geostrophic circulation given the weak lateral stratification. The surface mixed-layer in summer is formed by solar heating and wind-forcing and deepens over the summer period, with very weak gradients in the top 10 m. However, there is a strong, often nearly linear, gradient in temperature, salinity, and density from the base of the surface mixed-layer (10 m) to at least 25 m depth (Fig. S1). Consequently, there is a strong density gradient and a clear boundary between the well-mixed surface waters (0 to 10 m) and the stratified near-bottom water from 10 to at least 25 m below the surface. St. George’s Bay was described in greater detail in a previous study [24].

## Methods

### Field sampling

**Figure 1:**
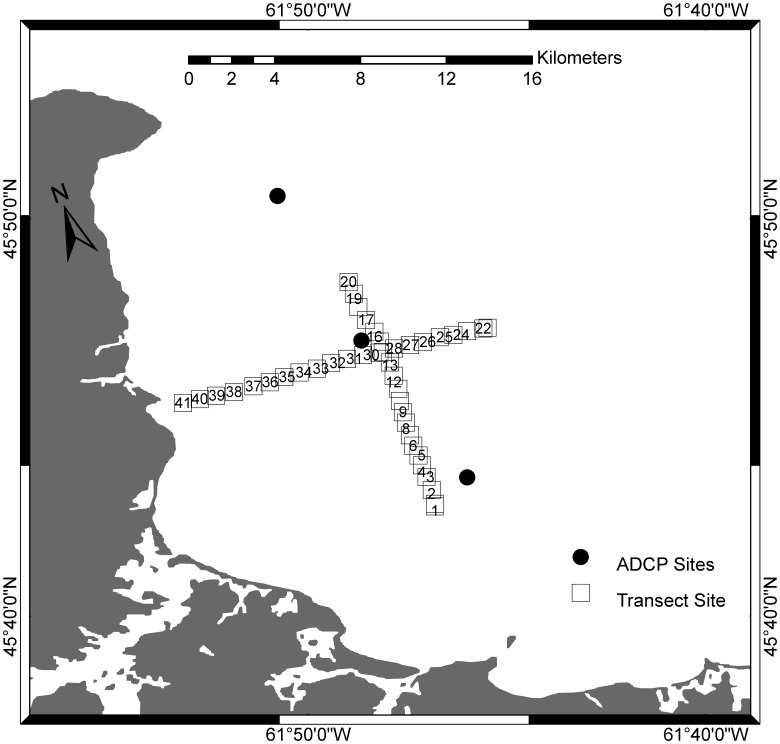
Map of St. George’s Bay, Nova Scotia, Canada indicating sites along 2 perpendicular ∼10-km transects which were sampled continuously (one larval sample every ∼500 m for 5 min) at 3 m depth, with a 200-µm plankton ring net (0.75 m diameter). The entire water column was profiled with a CTD between larval samples. The locations of the ADCP moorings are also shown.

Larval abundance and biological and physical variables were sampled in St. George’s Bay, Nova Scotia, on 15 Aug 2009. No specific permissions were required for these locations/activities. The field study did not involve endangered or protected species. The sampling area was from 45.713 to 45.804°N and from 61.882 to 61.759°W, specific site coordinates can be found in the Data S1. Two 10-km transects (Fig. 1) were sampled in contiguous 500-m transects for 5 min each at 3 m depth with a 200-µm plankton ring net (0.75-m diameter). For each tow, we repositioned the boat to ensure that the coordinates of the start position of the tow matched the end position of the previous tow. The net was towed at ∼1.7 m s^−1^ for 5 min and the volume of filtered water was quantified using a flowmeter. Temperature, conductivity, pressure and fluorescence were measured through the entire water column by doing vertical casts with a Seabird 25 Conductivity-Temperature-Depth (CTD) recorder, and an attached SCUFA fluorometer. CTD casts were done between plankton tows and at both ends of the transects. Each transect consisted of 20 plankton samples to quantify larval abundance, and 21 CTD casts. The CTD recorded at 1 Hz, but the data from 1–5 m depth (which was always in the surface mixed layer at all sites) were averaged to provide a representative estimate of the physical variables at 3 m. The depth of the mixed layer was calculated by determining the shallowest depth at which the density differences between consecutive measurements exceeded 2 standard deviations of all density differences between consecutive measurements of the entire density profile between 1–5 m. Each transect took 6 h to complete and was timed so that high tide (which coincided with high noon) would occur between transects. By ensuring that transects were sampled during a single phase of the tidal cycle (and light cycle), any effect of tidal or diel vertical migration would be of the scale of the entire transect (10 km). The first transect was sampled from South to North (low to high tide) and the second transect was sampled from East to West (high to low tide). Using a net of this mesh size may under-estimate abundance of small larvae (<200 µm). However, it is a necessary compromise in this multi-species study to allow capture of a wide range of larval types at sufficient numbers (e.g. very abundant but small gastropods to larger but rare decapods). All plankton samples were preserved in 95% ethanol and larvae were identified and enumerated under a Nikon SMZ 1500, as described in Lloyd et al. [25]. Samples were split into subsamples using a Folsom plankton splitter. Samples from 8 of the stations were split to 1/64 of the original volume and all subsamples were processed. Based on those samples, we determined that at least 20 individuals of each species were required to obtain an estimate of abundance that was within 5% of the true sample abundance. The remainder of the samples were split to 1/128–1/1 to ensure that ≥20 individuals of the most abundant species (*Margarites* spp., *Astyris lunata*, *Mytilus* spp., *Electra pilosa* and *Cancer irroratus*) were enumerated. Individual larval species have also been combined into broader taxonomic groupings to generalize the interpretation of results.

Three 600 kHz Teledyne RDI Workhorse Sentinel Acoustic Doppler Current Profilers (ADCP) were deployed on the seafloor, sampling the full water column in 1-m depth bins every 20 min from11 July to 22 Aug 2009 (Fig. 1). We only included the horizontal velocities in the bin centered at 3 m depth in our analyses. The reliability of the current velocities at 3 m was assessed by plotting the variance in current velocities for each 1-m depth bin for the entire water column; the variance in the 2.5–3.5 m bin was not greater than that in the mixed layer below it (∼4–12 m). Cross-correlation and coherence-squared analyses were applied to the current data collected by the 3 ADCP in Aug 2009. For the spatial/temporal analyses, the data record was truncated to 4 days (12–15 Aug) to reflect recent hydrodynamic conditions that may have affected larval distributions at the time scale of our study. The mean horizontal speed over the week preceding the study was weak, 28.6 mm s^−1^ (0.1 km h^−1^), corresponding to a passive particle being transported roughly10 km (scale of larval sampling) over 4 days.

### Data analyses

We examined the relationships among abundances of different larval groups or species, as well as the relationship between the abundance of each larval group or species and each physical variable (temperature, salinity, fluorescence, density, depth of the fluorescence maximum and depth of the mixed layer) using Pearson correlations. The logarithm (base 10) of larval abundance data was used for all statistical tests because it improved the normality of count data [26].

Since this study was designed to examine small scale patterns specifically, we removed large scale trends in larval abundance by linearly regressing larval abundance through space and using the residuals for the spatial analyses. The smaller scale variability was still within the residuals, but the large scale variability (possibly related to vertical migration, sampling design, etc.) was removed. To quantify spatial and temporal autocorrelation, the residuals of larval abundances (to remove large scale variables) and the physical variables were analysed using Moran’s I calculated as:
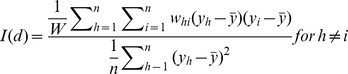
(1)


In this equation, *y_h_* and *y_i_* are the residual values of the variable of interest (abundance or physical parameter) at sites *h* and *i*, *d* is the distance class, *w_hi_* are the weights which take the value of 1 when sites *h* and *i* are at distance *d* or are equal to 0 otherwise, and *W* is the sum of those weights [27]. Moran’s I is a spatial autocorrelation statistic that takes on a null value when no autocorrelation is detected and varies from 1 to −1, indicating positive and negative spatial autocorrelation, respectively. Spatial lags were calculated for 12 bins with equal numbers of site pairs in each bin (n = 15–16 and 17–18, for larval abundances and physical variables, respectively) to allow bins to have the same statistical power. For the velocity data from the ADCP, temporal lags were calculated for 15 bins (n = 2755) to maintain a resolution similar to that of the spatial autocorrelation. To allow comparisons between the two, the temporal autocorrelation was converted to spatial autocorrelation by multiplying the time lag with the mean speed measured by the ADCP from 12–15 Aug (32.9 and 22.5 mm s^−1^, in the S-N and W-E directions, respectively). All autocorrelation analyses were done in SAM v4.0 [28].

For consistency and comparability, we chose to analyse all spatial and temporal patterns using Moran’s I. This metric was chosen because all data series except current velocity are relatively short, making conventional spectral analysis difficult (very coarse resolution). Additionally, with Moran’s I, the width of the distance or time bins can be customized to allow the use of the same number of variable pairs for each data point.

Patterns in the differences in physical variables among sites were explored using Nonmetric Multidimensional Scaling (nMDS). The nMDS plot was created using the “metaMDS” function in the “vegan” package [29] in R 2.14.1 [R Core 30]. The nMDS plot was then rotated in order for the first dimension (NMDS1) to be parallel to the abundance of each larval group, using “MDSrotate”. This function rotates the nMDS plot so that the dispersion of points of the abundance of a particular larval group is highest along NMDS1. This rotation allowed the visualization of the relationship among sites that were physically similar (a single water mass) or dissimilar (different water masses), and larval abundance.

## Results

### Larval distributions

**Figure 2:**
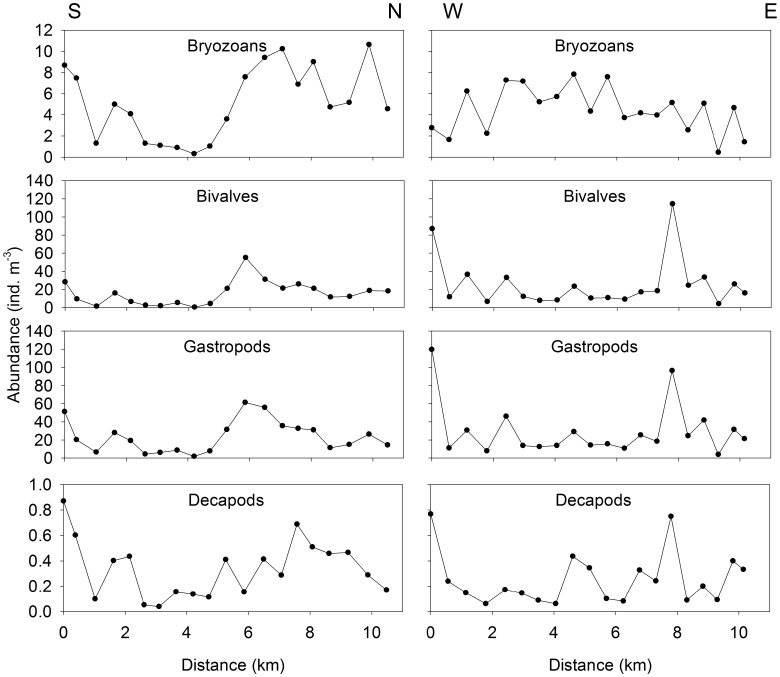
Larval abundance from North to South and West to East transects in St. George’s Bay, Nova Scotia, Canada, which were sampled continuously (one sample every ∼500 m for 5 minutes) at 3 m depth with a 200-µm plankton ring net (0.75 m diameter).

**Figure 3:**
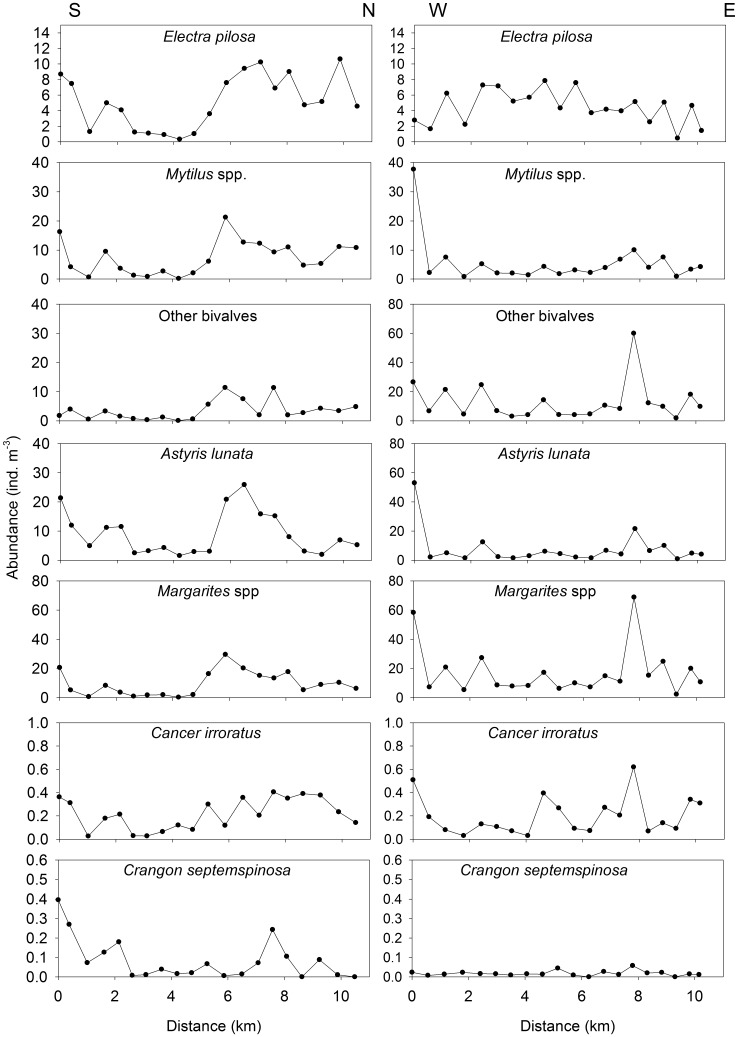
Larval abundance from North to South and West to East transects in St. George’s Bay, Nova Scotia, Canada, which were sampled continuously (one sample every ∼500 m for 5 minutes) at 3 m depth with a 200-µm plankton ring net (0.75 m diameter).

Collected larvae were categorized as bryozoans, bivalves, gastropods or decapods (Tab. 1). The bryozoans consisted almost entirely of *Electra pilosa*, whereas the bivalves were largely un-identifiable to species, but the most abundant identifiable genus was *Mytilus* spp. The gastropods consisted of *Margarites* spp. and *Astyris lunata*, and the decapods were mostly *Cancer irroratus*. In the S-N transect, the most striking feature in larval distribution of taxonomic groups was a peak in abundance at 5.8 km for both the bivalves and gastropods, and at the same location, an increase in abundance of bryozoan larvae (Fig. 2). At the species level, the patterns were similar except that the peak at 5.8 km was not as pronounced for the ‘other bivalves’ (Fig. 3). In the W-E transect, peaks in the abundance of bivalves, gastropods and decapods occurred at 0 km (closest to shore) and 7.8 km (Fig. 2). At the species level, the pattern was similar, but the peak at 7.8 km was less pronounced for *Mytilus* sp. and *A. lunata*, and there are no large peaks in the distribution of *Crangon septemspinosa* (Fig. 3).

**Table 1:** Mean, minimum and maximum abundance (individuals m^−3^) of larval taxonomic groups, during plankton sampling in St. George’s Bay, Nova Scotia, Canada, in Aug 2009.

A) Abundance							
Bryozoans		Bivalves		Gastropods		Decapods	
Mean	4.80		20.6		26.0		0.30
Minimum	0.32		0.46		1.92		0.04
Maximum	10.6		114		119		0.87
**B) Composition**							
**Species**	**%**	**Species**	**%**	**Species**	**%**	**Species**	**%**
*Electra pilosa*	99.9	*Mytilus* spp.	34.6	*Margarites* spp.	49	*Cancer irroratus*	71.7
*Membranipora membranacea*	0.1	*Modiolus modiolus*	14.6	*Astyris lunata*	32.5	*Crangon septemspinosa*	16.4
		*Anomia simplex*	5.4	*Diaphana minuta*	6.2	*Neopanopeus sayi*	8
		other	45.4	*Crepidula spp.*	4.6	*Carcinus maenas*	3.9
				*Arrhoges occidentalis*	4.1		
				*Bittiolum alternatum*	3.1		
				other	0.5		

Proportional species composition for each group is also shown.

The abundances of bivalves and gastropods were highly correlated with one another, while the correlation between the abundance of bryozoans and decapods was much weaker (Tab. 2). Intermediate correlation coefficients were obtained for the remaining pairs of taxonomic groups (Tab. 2). The abundances of bivalves and gastropods were also highly correlated at the species level, except for *A. lunata* (Tab. 3). Additionally, the abundance of *C. irroratus* was significantly correlated with that of all other species whereas that of *C. septemspinosa* was only correlated with *E. pilosa* and *A. lunata.* In general, there were more correlations between the physical variables and the abundance of bryozoans, gastropods and bivalves than for decapods (Tab. 4). Interestingly, all species were negatively correlated with fluorescence (in most cases significantly) and positively correlated with salinity (not always significantly) except *C. septemspinosa,* which was significantly negatively correlated with the depth of the fluorescence maximum. Bryozoan and bivalve abundance was significantly correlated with both fluorescence and salinity. Overall, fluorescence and salinity had the highest number of significant correlations with the abundance of all groups and species (Tab. 4).

**Table 2:** Pearson correlation examining the relationship in abundance for pairs of taxa: bryozoans (Bz), bivalves (Bv), gastropods (Gp) and decapods (Dp).

A)	Bz	Bv	Gp	Dp
Bz	1	**0.646**	**0.684**	**0.429**
Bv	**<0.0001**	1	**0.943**	**0.567**
Gp	**<0.0001**	**<0.0001**	1	**0.654**
Dp	**0.0057**	**0.0001**	**<0.0001**	1
**B)**	**Bz**	**Bv**	**Gp**	**Dp**
Bz	1	0.231	**0.317**	**0.402**
Bv	0.1514	1	**0.929**	**0.556**
Gp	**0.0464**	**<0.0001**	1	**0.649**
Dp	**0.0102**	**0.0002**	**<0.0001**	1

Relationships were assessed for both the logarithm (base 10) of the abundances variables (A) and the residuals of the abundances from the large scale spatial regression (B). The upper half of the matrix indicates the correlation coefficients for log_10_ (x+1) where x is larval abundance, lower half indicates the p-value. Statistically significant correlations are indicated in bold.

**Table 3:** Pearson correlation examining the relationship in abundance for pairs of species: *Electra pilosa* (Bz1), *Mytilus* spp. (Bv1), Other bivalves (Bv2), *Astyris lunata* (Gp1), *Margarites* spp. (Gp2), *Cancer irroratus* (Dp1), *Crangon septemspinosa* (Dp2).

A)	Bz1	Bv1	Bv2	Gp1	Gp2	Dp1	Dp2
Bz1	1	**0.631**	**0.419**	**0.491**	**0.668**	**0.405**	0.302
Bv1	**<0.0001**	1	**0.506**	**0.811**	**0.779**	**0.602**	0.279
Bv2	**0.0072**	**0.0009**	1	**0.435**	**0.853**	**0.480**	−0.142
Gp1	**0.0013**	**<0.0001**	**0.0051**	1	**0.636**	**0.557**	**0.432**
Gp2	**<0.0001**	**<0.0001**	**<0.0001**	**<0.0001**	1	**0.592**	0.073
Dp1	**0.0094**	**<0.0001**	**0.0017**	**0.0002**	**0.0001**	1	**0.365**
Dp2	0.0583	0.0807	0.3811	**0.0053**	0.6548	**0.0205**	1
**B)**	**Bz1**	**Bv1**	**Bv2**	**Gp1**	**Gp2**	**Dp1**	**Dp2**
Bz1	1	0.300	0.105	0.240	0.257	**0.359**	**0.391**
Bv1	0.0602	1	**0.341**	**0.905**	**0.692**	**0.479**	0.311
Bv2	0.5187	**0.0314**	1	**0.445**	**0.881**	**0.523**	−0.084
Gp1	0.1350	**<0.0001**	**0.0040**	1	**0.731**	**0.480**	**0.372**
Gp2	0.1093	**<0.0001**	**<0.0001**	**<0.0001**	1	**0.609**	0.065
Dp1	**0.0230**	**0.0018**	**0.0005**	**0.0017**	**<0.0001**	1	**0.315**
Dp2	**0.0126**	0.0506	0.6069	**0.0181**	0.6883	**0.0477**	1

Relationships were assessed for both the logarithm (base 10) of the abundances (A) and the residuals of the abundances from the large scale spatial regression (B). The upper half of the matrix indicates the correlation coefficients for log_10_ (x+1) where x is larval abundance, lower half indicates the p-value. Statistically significant correlations are indicated in bold.

**Table 4:** Pearson correlation coefficients examining the relationship among physical variables of the water column and abundance of A) taxonomic groups [bryozoans (Bz), bivalves (Bv), gastropods (Gp) and decapods (Dp)] and B) species [Electra pilosa (Bz1), Mytilus spp. (Bv1), Other bivalves (Bv2), Astyris lunata (Gp1), Margarites spp. (Gp2), Cancer irroratus (Dp1), Crangon septemspinosa (Dp2)] from 15 Aug 2009 for sampling depths of 3 m (n = 20).

Physical variables and logarithm (base 10) of abundance					
A)	Temperature	Salinity	Fluorescence	Z (F_max_).	Mixed Layer
Bz	−0.267	**0.637**	−**0.662**	0.096	−**0.445**
	(0.2553)	**(0.0025)**	**(0.0015)**	(0.6866)	**(0.0491)**
Bv	−0.194	**0.479**	−**0.593**	0.237	−0.281
	(0.4116)	**(0.0327)**	**(0.0058)**	(0.3134)	(0.2294)
Gp	−0.053	0.381	−0.431	0.125	−0.120
	(0.8234)	(0.0971)	(0.0580)	(0.5993)	(0.6153)
Dp	0.160	0.274	−0.414	−0.267	−0.076
	(0.5009)	(0.2429)	(0.0695)	(0.2555)	(0.7489)
**B)**	**Temperature**	**Salinity**	**Fluorescence**	**Z (F_max_)**	**Mixed Layer**
Bz1	−0.266	**0.637**	−**0.662**	0.095	−0.444
	(0.2574)	**(0.0025)**	**(0.0015)**	(0.6890)	(0.0496)
Bv1	−0.224	**0.508**	−**0.600**	0.186	−0.310
	(0.3434)	**(0.0223)**	**(0.0052)**	(0.4320)	(0.1841)
Bv2	−0.300	**0.491**	−**0.532**	0.235	−0.300
	(0.1987)	**(0.0280)**	**(0.0158)**	(0.3192)	(0.1995)
Gp1	0.110	0.557	−0.223	−0.041	0.098
	(0.6456)	(0.2740)	(0.3448)	(0.8643)	(0.6795)
Gp2	−0.119	0.427	−**0.573**	0.268	−0.245
	(0.6178)	(0.0602)	**(0.0082)**	(0.2537)	(0.2974)
Dp1	−0.056	**0.461**	−**0.651**	0.032	−0.352
	(0.8140)	**(0.0409)**	**(0.0019)**	(0.8927)	(0.1275)
Dp2	0.429	−0.093	0.022	−**0.615**	0.318
	(0.0594)	(0.6952)	(0.9281)	**(0.0039)**	(0.1722)

Relationships were assessed for the logarithm (base 10) of the abundances and the physical variables. The value in brackets indicates the p-value. Statistically significant correlations are indicated in bold.

When large scale spatial gradients were removed from the abundances of larvae and from the physical variables through spatial regression, the patterns were similar, but the relationship with fluorescence was stronger (Tab. 5). The abundance of *E. pilosa* and *A. lunata* was significantly correlated with fluorescence, and that for all other species abundance was also significantly negatively correlated with fluorescence except ‘other bivalves’ (Tab. 5). Lower fluorescence corresponded to a high larval abundance at the local scale (<10 km).

**Table 5:** Pearson correlation coefficients examining the relationship among physical variables of the water column and abundance of A) taxonomic groups [bryozoans (Bz), bivalves (Bv), gastropods (Gp) and decapods (Dp)] and B) species [Electra pilosa (Bz1), Mytilus spp. (Bv1), Other bivalves (Bv2), Astyris lunata (Gp1), Margarites spp. (Gp2), Cancer irroratus (Dp1), Crangon septemspinosa (Dp2)] from 15 Aug 2009 for sampling depths of 3 m (n = 20).

Residuals of physical variables and residuals of abundance					
A)	Temperature	Salinity	Fluorescence	Z (F_max_)	Mixed Layer
Bz	−0.151	**0.601**	−**0.708**	−0.139	−0.331
	(0.5253)	**(0.0051)**	**(0.0005)**	(0.5596)	(0.1541)
Bv	0.254	0.273	−**0.510**	0.202	0.010
	(0.2794)	(0.2238)	**(0.0217)**	(0.3920)	(0.9680)
Gp	0.209	0.360	−**0.581**	0.110	−0.030
	(0.3768)	(0.1189)	**(0.0073)**	(0.6454)	(0.8996)
Dp	−0.243	0.331	−**0.645**	−0.270	−0.235
	(0.3021)	(0.1546)	**(0.0022)**	(0.2498)	(0.3196)
**B)**	**Temperature**	**Salinity**	**Fluorescence**	**Z (F_max_)**	**Mixed Layer**
Bz1	−0.151	**0.601**	−**0.708**	−0.140	−0.329
	(0.5259)	**(0.0051)**	**(0.0005)**	(0.5547)	(0.1569)
Bv1	0.135	0.328	−**0.559**	0.009	−0.071
	(0.5698)	(0.1586)	**(0.0104)**	(0.9710)	(0.7677)
Bv2	0.101	0.257	−0.430	0.202	0.033
	(0.6709)	(0.2741)	(0.0586)	(0.3925)	(0.8911)
Gp1	0.203	**0.520**	−**0.705**	−0.009	−0.075
	(0.3904)	**(0.0189)**	**(0.0005)**	(0.9701)	(0.7547)
Gp2	0.269	0.277	−**0.528**	0.178	0.015
	(0.2519)	(0.2378)	**(0.0166)**	(0.4625)	(0.9505)
Dp1	−0.192	0.327	−**0.608**	−0.108	−0.302
	(0.4165)	(0.1599)	**(0.0044)**	(0.6489)	(0.1952)
Dp2	−0.306	0.319	−**0.627**	−**0.488**	−0.090
	(0.1893)	(0.1708)	**(0.0031)**	**(0.0290)**	(0.7061)

Relationships were assessed for the residuals of the abundances and residuals of the physical variables from the large scale spatial regression. The value in brackets indicates the p-value. Statistically significant correlations are indicated in bold.

**Figure 4:**
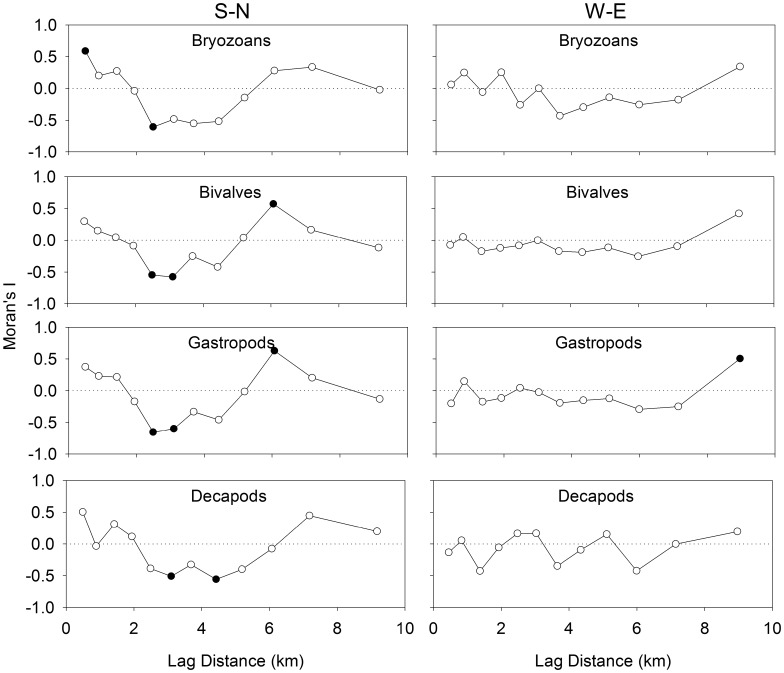
Spatial analysis of the abundance for each of 4 larval groups (*Electra pilosa, Mytilus* spp., Other bivalves, *Astyris lunata, Margarites* spp., *Cancer irroratus, Crangon septemspinosa*) using Moran’s I. Positive and negative values of Moran’s I indicate positive and negative spatial autocorrelation, respectively. Filled circles indicate significant spatial autocorrelation at that scale.

**Figure 5:**
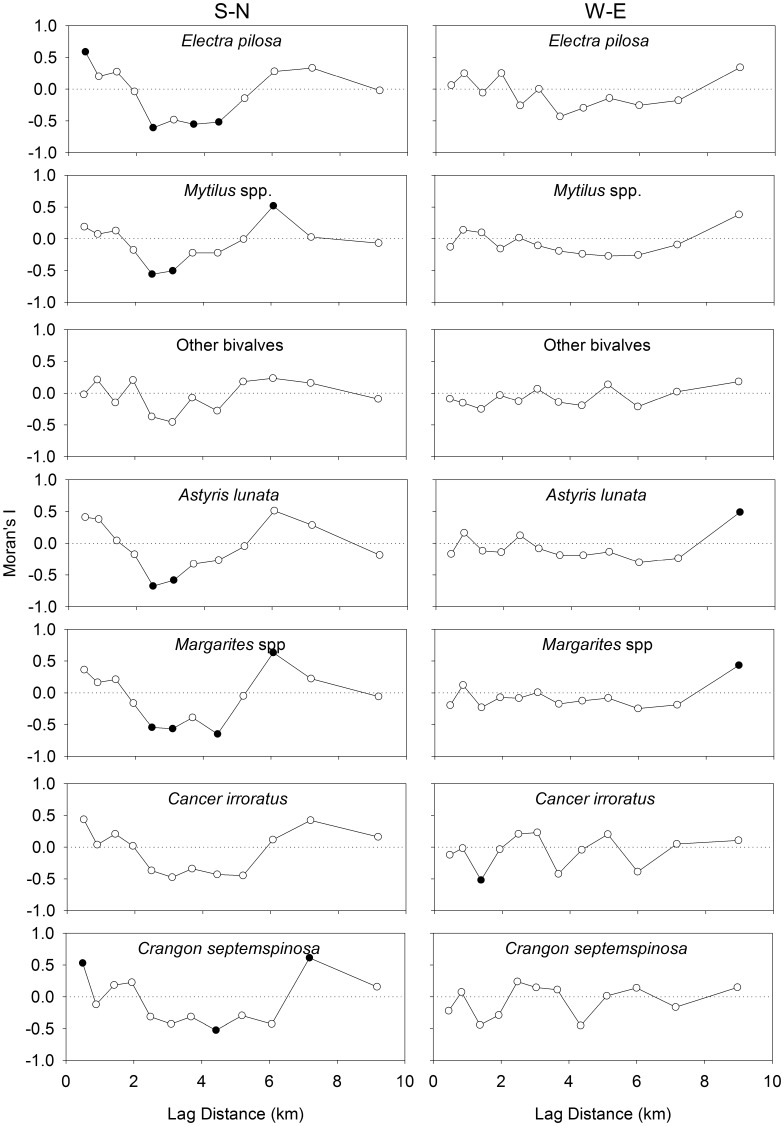
Spatial analysis of the abundance for each of 7 larval species (gastropods, bivalves, bryozoans, and decapods) using Moran’s I. Positive and negative values of Moran’s I indicate positive and negative spatial autocorrelation, respectively. Filled circles indicate significant spatial autocorrelation at that scale.

On the S-N transect, significant positive spatial autocorrelation of larval abundance was observed at the smallest observable scale of 0.5 km for bryozoans, and at 7.2 km for the gastropods and bivalves (Fig. 4). Significant negative autocorrelation of abundance was observed at 2.5 km for bryozoans, 2.5 and 3.0 km for bivalves and gastropods, and 3.0 and 4.3 km for decapods. In the W-E transect, no statistically significant spatial autocorrelation of larval abundance was observed for any taxonomic group, except gastropods at 9.2 km. Unsurprisingly, a similar pattern is present at the species level (Fig. 5). In the S-N transect, all species except ‘other bivalves’ and *C. irroratus* had significant negative spatial autocorrelation between 2.5 and 4.3 km and positive spatial autocorrelation between 6 and 7.2 km.

### Distribution of physical variables in the transects

**Figure 6:**
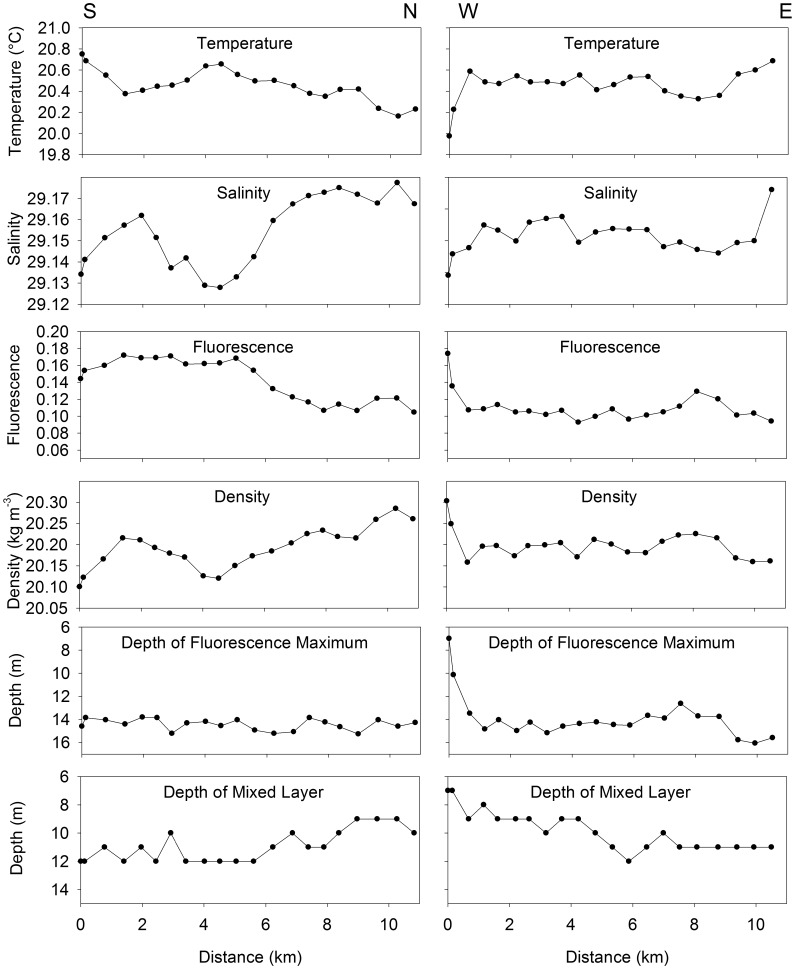
Physical variables in the water column of North to South and East to West transects in St. George’s Bay, Nova Scotia, Canada which were sampled once every ∼500 m. Data represents the average for the water column from 1–5 m depth.

**Figure 7:**
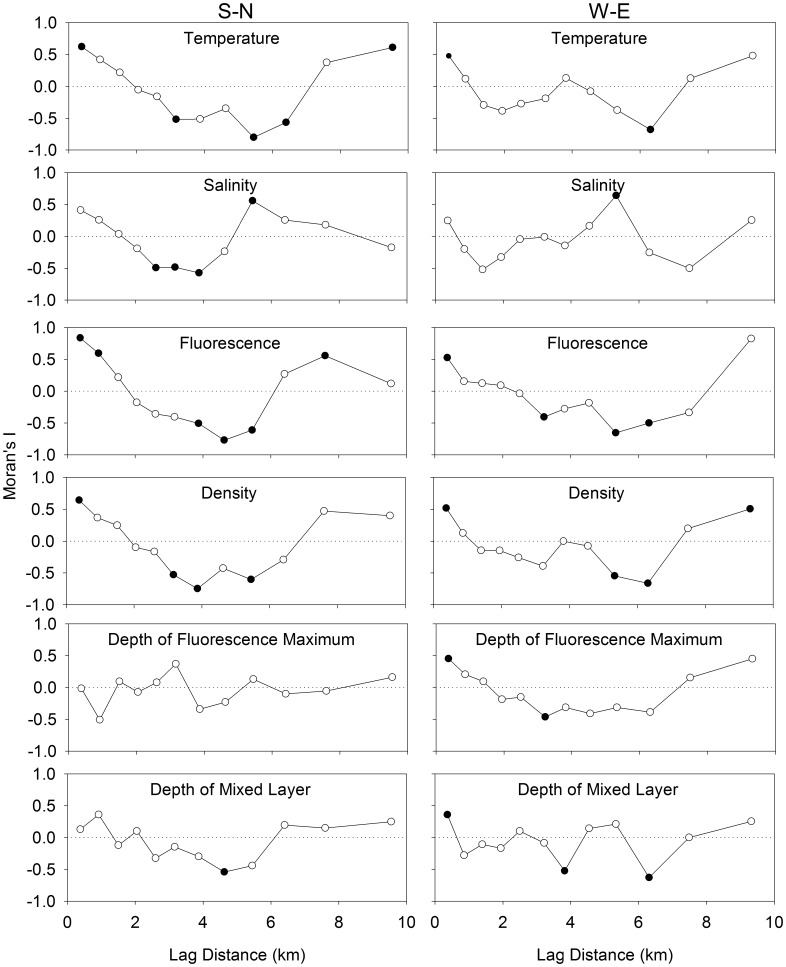
Spatial analyses of the physical variables using Moran’s I. Positive and negative values of Moran’s I indicate positive and negative spatial autocorrelation, respectively. Filled circles indicate significant spatial autocorrelation at that scale.

In the S-N transect, there was a large increase in salinity (front) at ∼6 km, with a corresponding decrease in fluorescence (Fig. 6). In the W-E transect, the most identifiable feature was the low temperature and salinity combined with high fluorescence, density and depth of fluorescence maximum at 0 km, which was closest to shore. Significant positive spatial autocorrelation was observed in temperature, density and fluorescence at the smallest observable scale of 0.4 km on both transects, and in the depth of the fluorescence maxima and mixed layer in the S-N transect (Fig. 7). Additionally, in the S-N transect, 3 out of 6 of the variables were significantly negatively autocorrelated at 3.2, 3.9 and 5.5 km. In the W-E transect, more than one of the physical variables were significantly negatively autocorrelated at 3.2, 5.3 and 6.3 km. Salinity was significantly positively autocorrelated at 5.5 km in the S-N transect, and at 5.3 km in the W-E transect.

**Figure 8:**
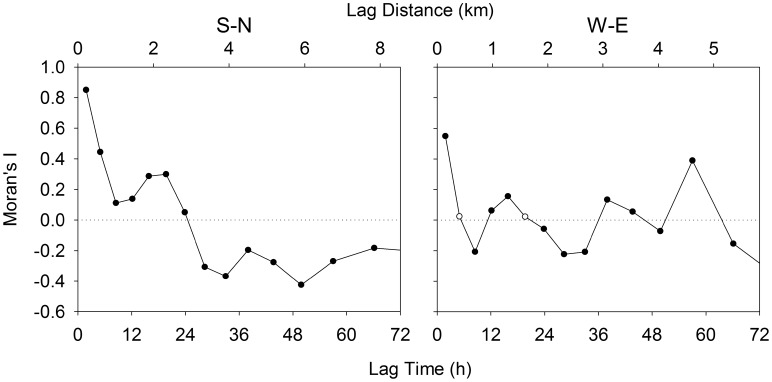
Temporal analyses of horizontal current velocities using Moran’s I. Positive values of Moran’s I indicate positive spatial autocorrelation and vice versa. Filled circles indicate significant spatial autocorrelation at that scale. Equivalent lag distances were calculated by multiplying the time lag by the mean speed (see Methods).

The cross-correlation in current velocity among the ADCP moorings was just under 0.6 for moorings separated by 3–4 km, and 0.2–0.3 for moorings separated by 5–6 km. Only at tidal periods was coherence squared greater than the significance threshold of 0.3 (just below 0.6 for neighbouring moorings). The current velocity data showed significant temporal autocorrelation at most time lags because of the large sample size (Fig. 8). An important difference in the pattern of autocorrelation between directions is that the peak at 8.5 h is positive in the S-N direction; therefore, the first negative peak in autocorrelation is at 33 h in the S-N direction and 8.5 h in the W-E direction. After being converted to distance by multiplying by the mean speed, these time lags correspond to 3.9 and 0.7 km, in the S-N and W-E direction, respectively. Spectral analysis (Daigle, unpublished data) also indicated that the dominant peak in the periodogram for the S-N transect is at a lower frequency than in W-E transect. This suggests that the oscillations along the S-N transect occur over a longer time period, translating into larger spatial scales, than in the W-E direction.

### Biophysical interactions

**Figure 9:**
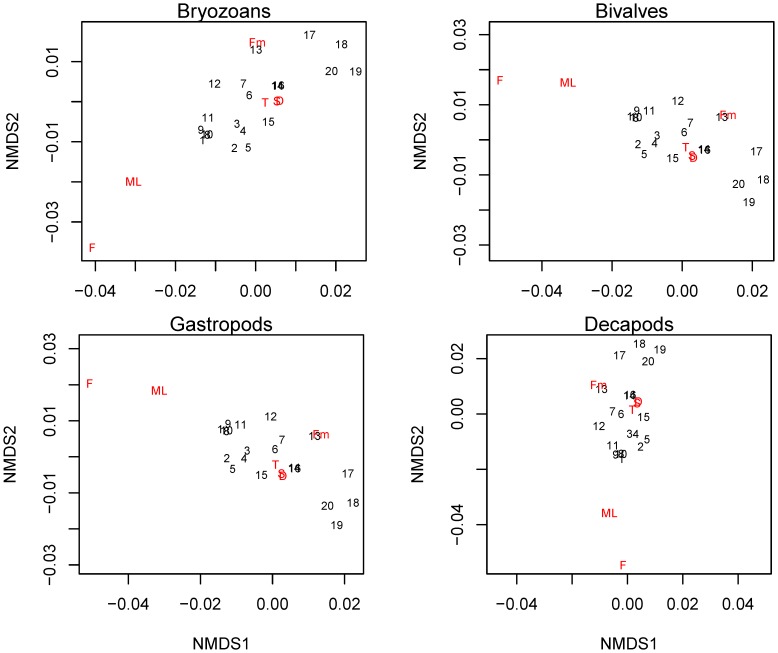
Ordination from nonmetric multidimensional scaling of the sampling sites based on physical variables. The ordinations were rotated so that NMDS1 is parallel to an external variable (abundances of bryozoans, bivalves, gastropods and decapods). Larval abundance increases from left to right. Numbers indicate the location of the site in nMDS ordination space and the letters represent the physical variables of the water column [temperature (T), salinity (S), fluorescence (F), density (D), depth of the fluorescence maximum (Fm) and depth of the mixed layer (ML)]. The letter’s distance from the origin represents the weight of that factor in the ordination space and the values for the physical variables increase from the origin to the letters indicating that variable. Stress = 0.0486.

The dispersion of sites classified by their physical variables in the rotated nMDS plots was relatively parallel to NMDS1 for bryozoans, bivalves and gastropods compared to NMDS1 for the decapods (Fig. 9). The loadings for the physical variables were oriented perpendicular to the gradient of larval abundance for the decapods. For the other taxonomic groups, the loadings of the physical variables varied in parallel to the larval gradient. Fluorescence and the depth of the mixed layer had the largest influence on the separation of the sites indicated by their letter symbols being furthest from the origin.

## Discussion

In this study, as on the scale of the entire bay [24], the distributions of gastropods and bivalves were most similar to one another, and those of bryozoans and decapods were most different from one another. Gastropods and bivalves are similar morphologically (*i.e.* calcareous shells), have similar swimming ability, and both groups display vertical migration [25], [31]. If different types of larvae behave similarly or have similar early life history strategies, then they will likely aggregate in the same types of oceanographic features [10], [32], [33]. Conversely, contrasting life strategies result in very different dispersal properties, and consequently, different larval distributions. Bryozoans are among the slowest swimmers [31], [34], and decapods are among the fastest of the invertebrate larvae [35], [36]. It is well established that larval behaviour can be species- and even stage-specific, which can lead to differences in patterns of horizontal distribution [18], [22], [25], [37]. In this study, species specific behaviour may be observed in *C. septemspinosa*. However, the similarity in larval distribution among most species is suggestive of common mechanisms of pattern formation.

The consistent relationship between bryozoan abundance and salinity suggests that bryozoans were being advected like a passive tracer such as salinity. We found that the abundance of bryozoans was negatively correlated with fluorescence although others have found that the peak in the vertical bryozoan abundance was associated with the fluorescence maximum [25]. Similarly, for all species, lower fluorescence corresponded to a high larval abundance at the local scale (<10 km). Given the relatively small horizontal differences in fluorescence compared to vertical ones, the association with lower fluorescence likely reflects an association with a particular water mass with low fluorescence rather than with food availability. Additionally, we found that the abundance of bryozoans, gastropods and bivalves had a stronger relationship than the decapods with the physical variables in multivariate space. While the abundance of *C. irroratus* had generally weaker relationship with physical variables than that of bryozoan, bivalve and gastropod species, the pattern differed for *C. septemspinosa*. The negative relationship between the abundance of *C. septemspinosa* and the depth of the fluorescence maximum combined with the relatively weak relationship with the other physical variables, suggests that: either 1) larvae aggregated in areas where the fluorescence maximum is relatively shallower (*i.e.* available to them during the deep phase of their vertical migration); or 2) larval mortality was lower in these regions. However, the horizontal distribution for this species suggests that the larvae are largely retained in the southern portion of the bay.

For both the larvae (all groups, most species) and all the physical properties of the water column, except the fluorescence maximum, there was significant negative autocorrelation at distance lags between 2.5 and 5 km in the S-N direction. The first peak in negative autocorrelation of current velocity at time lags of 28 or 33 h corresponded to spatial lags of 3.3 to 3.9 km. While the peaks in autocorrelation of current velocity didn’t quite correspond with the M2 tidal cycle period (12.42 h), the offset in periodicity likely reflects an interaction between the tidal cycle and the diel cycle (24 h) of wind driven circulation. Given the scale of spatial autocorrelation in the W-E direction, it is not surprising that our transect sampling did not reveal patterns similar to the N-S direction. In the W-E direction, the first peak (at 8.5 h) in negative autocorrelation of the ADCP current velocities was as large as all other peaks. This indicates that the data were dominated by the signal that corresponds to that peak. Since this peak corresponds to a distance lag of 1 km, any spatial autocorrelation along the W-E transect would not have been properly resolved by the larval sampling at a frequency of 500 m. The method used to convert temporal to spatial autocorrelation in current velocity (multiplying the time lag with the mean speed), has similar assumptions to a progressive vector method which converts Eulerian observations into Lagrangian data. In both cases, it is assumed that the current is spatially uniform and only varies in time. This method is justified since there was stronger autocorrelation in the current velocities among the ADCPs that were closer together (3–4 km), indicating some spatial uniformity (coherence) of current velocities at this scale.

While it is well known that larval distribution can be affected by salinity, temperature, density or food availability [38]–[40], the observed variance in these variables in our study is biologically irrelevant. For example, a change in temperature of 0.075°C over a 500-m distance (which was the average thermal gradient) is unlikely to elicit a larval behavioural response. Such a gradient also should not have any direct consequences for growth or mortality. It is also possible that larval patches can result from predator-prey diffusive instabilities, where predator and prey disperse at different rates (possibly due to swimming ability) and the resulting predator-prey interactions can cause spatial heterogeneity in larval abundance [41]. In this case, larvae could be either the predator or the prey. However, diffusive instabilities are thought to occur at larger scales (10–100 km), and those instabilities do not explain the relationships among physical variables of the water column and the larval distributions.

We propose that the observed relationships between the abundance of larval groups and physical variables are the result of the non-uniform mixing of water masses. Given the weak mean currents (∼0.02 m s^−1^) it is clear that the residence time for larvae in St. George’s Bay can be quite long, on the order of days to tens of days, O(40 km/0.02 m s^−1^ ≈ 20 days), a result supported by numerical simulations (B. deYoung, unpublished data). This residence time is also very similar to the estimate of 15 d based on current meter observations in 1974–1975 (Petrie and Drinkwater, 1978). The relatively low cross-correlation in velocity among moorings suggests that large scale, coherent flow features in St. George’s Bay are relatively weak. The persistent tide (M_2_ tide is dominant, with a period of 12.42 hours) and the weak mean circulation generate an ideal dispersive environment. The long residence time and the weak spatial coherence in the velocity field at long periods and large spatial scales, combined with the regular tidal dispersion, twice daily at short spatial scales (km’s) will lead to significant dispersion in larval patterns [42]. By definition, flow structures with low coherence act to enhance mixing within a water-mass and coherent flow structures act to transport a water mass. We argue that the significant spatial coherence at tidal periods reduced advective diffusion (diffusion of larvae by means of advection as opposed to random diffusion, which is relatively constant) at that scale, and the low coherence at smaller scales enhances advective diffusion; spatial structures at those smaller scales will be erased while structure at the scale of the tidal period will be preserved.

We suggest that the scale of cyclical periodicity of coherent structures in current velocity (*i.e.* tidal period in this case) affects not only the physical properties of the water column, but also larval distributions. This effect of cyclical periodicity in current velocities should be particularly noticeable in the autocorrelation patterns of the poor swimmers (i.e. quasi-passive tracers), since their distribution is most related to the physical properties of the water column. Indeed, the peak in negative autocorrelation is lower for bryozoans than for decapods. The difference in patterns between the two transects suggests that the scale of cyclical periodicity in current velocities may play a directionally dependent role in the scaling of larval patchiness. Consequently, to properly resolve larval patchiness, biophysical models used to predict larval dispersal should at the very least resolve to the scale of the tidal excursion or the scale of the wind driven circulation, whichever is smallest.

Overall, we found significant negative autocorrelation at a similar spatial scale in all larval groups (most species) and all the physical properties of the water column, yet relatively few correlations between larval distributions and physical properties of the water column. This is not surprising since matching scales of negative spatial autocorrelation does not imply linear correlation between those variables. For example, a larval aggregation at an oceanographic front [32], [43] would be manifested as a larval patch centered on the boundary of two “physical” patches. If this scenario is repeated over space, we would detect consistent scales of spatial autocorrelation, yet no correlation between larval distributions and physical properties of the water column.

Our observations provide a snapshot of the conditions in the bay on that particular day, since sampling was conducted over a single 12-h period, and may not be representative of average conditions. However, observations from three different sampling periods at the scale of the entire bay also suggested that distributions of gastropods and bivalves were most similar to, and those of bryozoans and decapods were most different from one another [24]. Additionally, the negative relationship between fluorescence and larval abundance was remarkably consistent across taxa. We contend that the most parsimonious explanation for the similarity in scales of the spatial coherence in the tidal period, the larval distributions and the physical variables is that they are related due to the reduced advective diffusion at that scale and/or the advective-diffusive history of the water masses. In either case, the scale of the coherent structure (tides) is important in determining the patterns in larval distribution. Furthermore, we suggest that the scale of coherent structure in current velocities may be important in forming larval distribution patterns in other systems, which similarly to ours are not dominated by strong oceanographic features (e.g. estuarine plumes, upwelling events, or markedly different water masses). While our sampling design may limit our observations to depths of 3 m, it is reasonable to apply our suggested mechanism for scaling of larval patches to coherent structure in current velocities to other depths; the caveat would be that the spatial scale of the patches should scale to the local scale of coherent structures, which may not necessarily be ∼3 km.

The effects of the spatial distribution of the adult habitat and the influence of large scale hydrographic conditions (i.e. estuarine plume, eddies) on larval distributions [44]–[46] or small scale aggregating processes [10], [32] have been identified previously. However, these effects influence larval distributions at very large scales (>100 km), in locations with very different water masses, or at scales below the resolution of our study. Our study shows that small scale larval patchiness (<10 km) can exist even in areas of relatively homogenous physical properties. It is well established that the distribution of phytoplankton and holoplankton are affected by a variety of processes with multiple and overlapping scales [41], [47], [48], but the relative importance of all these processes on meroplankton is poorly known at present [10], [49], [50]. These studies have shown that larvae are not passive particles randomly drifting in the ocean. Their active interactions with the water column can affect their horizontal distribution at multiple scales from small-scale aggregations at fronts, to medium scale patches such as those observed, to large scale oceanographic features [1], [2], [22]; ultimately these larval behaviours affect larval dispersal.

Unlike holoplankton, the larvae of benthic invertebrates settle on the seafloor before taking their adult form, and the patchiness of larval distributions can affect the distributions of adults [49], [51], [52]. This effect can result from direct settlement of “patches”, but also indirectly by changing larval dispersal pathways through aggregations at fronts, advection along with water masses or vertical migration. We have shown that larval groups with similar life strategies (*e.g.* swimming ability) have similar distributions and that the weaker swimming larvae have a demonstrably tighter relationship with the physical properties of the water column, suggesting that they have dispersal properties similar to that of a passive tracer. Therefore, incorporating larval behaviour in biophysical models of larval transport would be more important in larvae with strong swimming abilities, whereas it may not be as important for weak swimmers. Additionally, we have shown that the scale of larval patchiness corresponds with that of both the physical variables of the water column and the tidal/diel periodicity in the current velocities. Factors such as larval swimming ability and the scale of coherent structures (*e.g.* tidal period), which determine larval distribution and patch size, will affect larval dispersal and ultimately the recruitment of benthic adults.

## Supporting Information

Figure S1 **Depth profiles of physical variables in the water column of N-S and W-E transects in St. George’s Bay, Nova Scotia, Canada, were sampled every ∼500 m.** White areas in the W-E transect represent the seafloor.
(TIFF)

Data S1 **Larval abundance (ind. m^3^) from North to South and West to East transects in St. George’s Bay, Nova Scotia, Canada, which were sampled continuously (one sample every ∼500 m for 5 minutes) at 3 m depth with a 200-µm plankton ring net (0.75 m diameter).** Physical variables in the water column were sampled once every ∼500 m. Physical variables data represents the average for the water column from 1–5 m depth.
(CSV)
