# Supplementary figures and images for: Fine-Scale Distribution and Spatial Variability of Benthic Invertebrate Larvae in an Open Coastal Embayment in Nova Scotia, Canada
Source: PLoS One. 2014 Aug 25;9(8):e106178. doi: 10.1371/journal.pone.0106178 (PMC4143338; doi:10.1371/journal.pone.0106178)

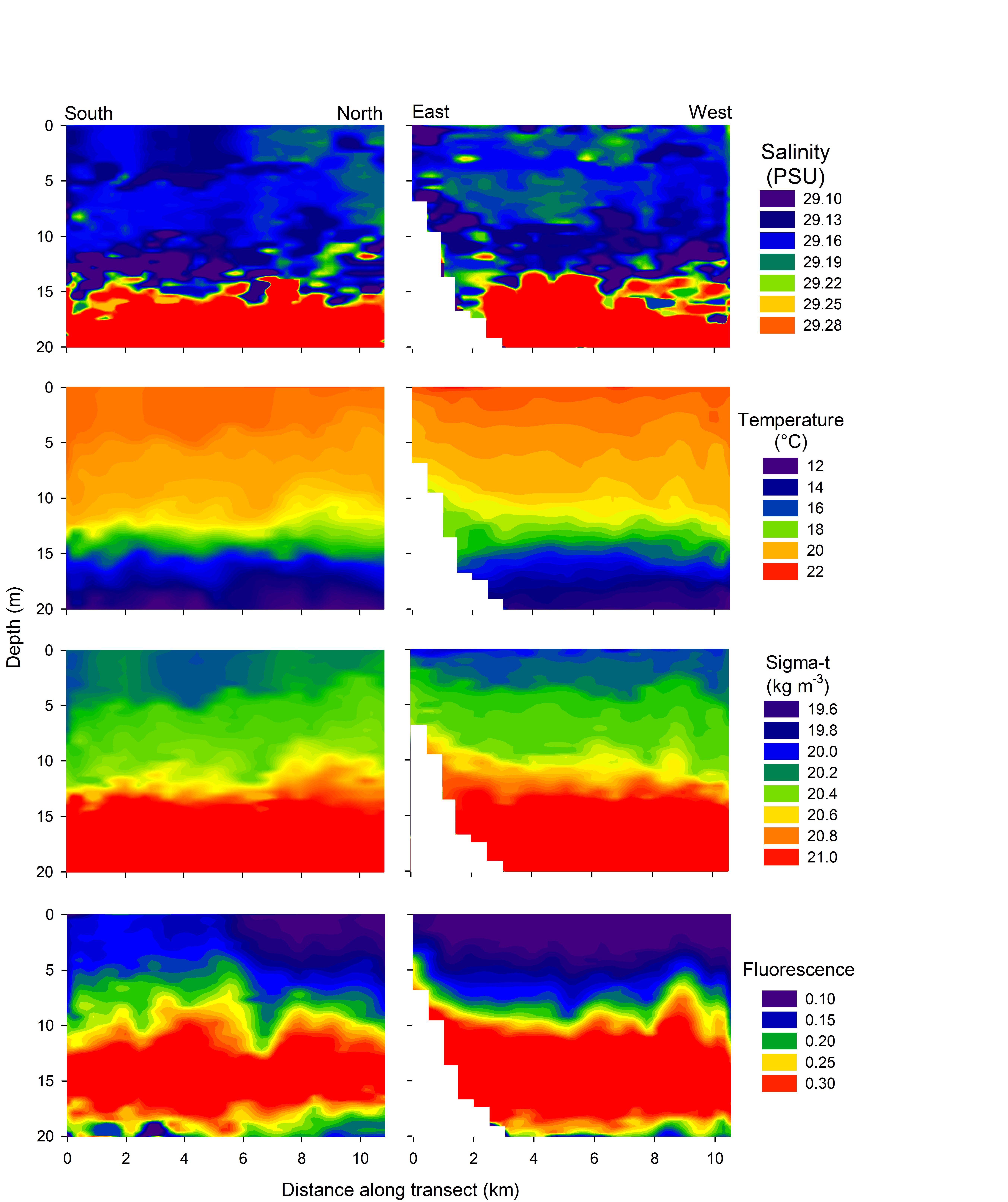

Supplement: Figure S1 — Depth profiles of physical variables in the water column of N-S and W-E transects in St. George’s Bay, Nova Scotia, Canada, were sampled every ∼500 m. White areas in the W-E transect represent the seafloor. (TIFF) [file pone.0106178.s001.tiff]
